# Early Mortality in Adults Initiating Antiretroviral Therapy (ART) in Low- and Middle-Income Countries (LMIC): A Systematic Review and Meta-Analysis

**DOI:** 10.1371/journal.pone.0028691

**Published:** 2011-12-29

**Authors:** Amita Gupta, Girish Nadkarni, Wei-Teng Yang, Aditya Chandrasekhar, Nikhil Gupte, Gregory P. Bisson, Mina Hosseinipour, Naveen Gummadi

**Affiliations:** 1 School of Medicine, Johns Hopkins University, Baltimore, Maryland, United States of America; 2 Bloomberg School of Public Health, Johns Hopkins University, Baltimore, Maryland, United States of America; 3 Johns Hopkins Clinical Trial Unit, Byramjee Jeejeebhoy Medical College, Pune, India; 4 School of Medicine, University of Pennsylvania, Philadelphia, Pennsylvania, United States of America; 5 School of Medicine, University of North Carolina, Chapel Hill, North Carolina, United States of America; Jiangsu University, China

## Abstract

**Background:**

We systematically reviewed observational studies of early mortality post-antiretroviral therapy (ART) initiation in low- and middle-income countries (LMIC) in Asia, Africa, and Central and South America, as defined by the World Bank, to summarize what is known.

**Methods and Findings:**

Studies published in English between January 1996 and December 2010 were searched in Medline and EMBASE. Three independent reviewers examined studies of mortality within one year post-ART. An article was included if the study was conducted in a LMIC, participants were initiating ART in a non-clinical trial setting and were ≥15 years. Fifty studies were included; 38 (76%) from sub-Saharan Africa (SSA), 5 (10%) from Asia, 2 (4%) from the Americas, and 5 (10%) were multi-regional. Median follow-up time and pre-ART CD4 cell count ranged from 3–55 months and 11–192 cells/mm^3^, respectively. Loss-to-follow-up, reported in 40 (80%) studies, ranged from 0.3%–27%. Overall, SSA had the highest pooled 12-month mortality probability of 0.17 (95% CI 0.11–0.24) versus 0.11 (95% CI 0.10–0.13) for Asia, and 0.07 (95% CI 0.007–0.20) for the Americas. Of 14 (28%) studies reporting cause-specific mortality, tuberculosis (TB) (5%–44%), wasting (5%–53%), advanced HIV (20%–37%), and chronic diarrhea (10%–25%) were most common. Independent factors associated with early mortality in 30 (60%) studies included: low baseline CD4 cell count, male sex, advanced World Health Organization clinical stage, low body mass index, anemia, age greater than 40 years, and pre-ART quantitative HIV RNA.

**Conclusions:**

Significant heterogeneity in outcomes and in methods of reporting outcomes exist among published studies evaluating mortality in the first year after ART initiation in LMIC. Early mortality rates are highest in SSA, and opportunistic illnesses such as TB and wasting syndrome are the most common reported causes of death. Strategies addressing modifiable risk factors associated with early death are urgently needed.

## Introduction

Ninety-five percent of the estimated 33.4 million HIV-infected adults live in low- and middle-income countries (LMIC) and approximately 7.1 million of these individuals have advanced HIV and are in need of antiretroviral treatment (ART) [1]. A massive global effort to provide ART is underway and if current trends continue, by 2015, 8 million HIV-infected persons will be receiving ART. ART has dramatically reduced morbidity and mortality worldwide [2]–[4]; however a >3-fold increased rate of mortality within the first 12 months post-ART initiation has been observed in LMIC compared to high-income countries [5].

A summary of cohorts of sub-Saharan African (SSA) settings previously identified a mortality estimate of 8%–26% at 12 months post-ART initiation [6]. However, this review did not include studies from Asia or the Americas, where substantial numbers of patients are also initiating ART. Furthermore, while it is clear that starting ART at higher CD4 counts will reduce mortality, many patients at ART clinics in LMIC continue to present with advanced HIV. Therefore, the objective of our study was to systematically review observational cohort data on early mortality, which we defined as death within 12 months post-ART initiation among persons in LMIC. We specifically wanted to measure the early mortality post-ART initiation in different regions, assess the relative causes of early mortality, and determine risk factors for mortality in these settings; such data are needed to inform global programs and global priorities for HIV care and treatment.

## Methods

### Search strategy and selection criteria

We searched Medline and EMBASE databases for articles published between January 1996, (when combination ART was heralded) and December 2010. *Keywords* used for the search strategy included “developing countries”, “Africa”, “South America”, “Asia”, “developing country”, “mortality”, “death”, “deaths”, “antiretroviral therapy”, “highly active”, “haart”, “anti-retrovirals”, “antiretrovirals”, “antiretroviral”, ”anti retroviral”, “highly active antiretroviral therapy” (see **table S1** for exact search strategy used). We limited the search to human studies and articles published in English. A total of 1776 potentially relevant citations were identified and compiled from both electronic databases; 875 articles remained after elimination of duplicates. Three reviewers (NG, GN, AC) screened titles and abstracts to capture potentially relevant studies and one reviewer (AG) resolved any discrepancies between them. Studies were included if they met the following criteria: a) study was in a LMIC as defined by the World Bank in 2008 {http://data.worldbank.org/about/country-classifications}, b) age of the participants was ≥15 years, c) participants were ART naïve at baseline or the authors gave data separately for naïve and non-naïve, d) mortality was reported within the first year after ART initiation and, e) size of the cohort was >50 participants. We excluded editorials, case reports, abstracts, letters, reviews, clinical trials, studies including only children and studies that focused on deaths due to a specific opportunistic infection. Full text review was completed by three independent reviewers (GN, WY and NG). Additional data were not obtained from study authors.

### Data extraction and analysis

For studies that met the inclusion criteria, data were extracted and results tabulated. The primary objective of the study was to measure early mortality post-ART initiation in different LMIC regions. The defined regions were LMIC in SSA, Asia, the Americas (South America, Central America and the Caribbean), and multi-regional (Africa, Asia and the Americas). We reported mortality data using the methods presented in each article including probability of survival using Kaplan-Meier curves; incidence rates; and proportions. Meta-analysis was done to estimate an overall and also a combined estimate of mortality rate within each region. Heterogeneity between the studies was examined using Cochran's Q and the I^2^ statistic [7]. The first step in getting the combined mortality rate was done by obtaining the number of deaths in individual studies. Most studies, that used Kaplan-Meier methods, summarized the number of deaths and the number in the risk set at each time point. For the few studies for which we did not have these data available, methods described in Parmar *et al* [8] and Whitehead & Whitehead [9] were used to estimate the number of deaths and lost to follow-up. If there was an evidence of variation between the studies, a random effects model was used to estimate the combined mortality rate and corresponding 95% confidence interval using standard methods [10], otherwise a fixed-effects model was used. The meta-analysis was summarized graphically using a Forest Plot. An analysis of publication bias was also performed using a Funnel Plot and Kendall's test [11]. All analyses were done using S-plus 8.1 (TIBCO, Palo Alto, CA) and StatsDirect (UK). A sensitivity analysis was done to estimate the bounds for the combined mortality rate. All lost-to-follow-up, for the upper bound were assumed to be dead (worst case scenario), and were assumed to be living for the lower bound (best case scenario). A DerSimonian & Liard estimate of mortality probability and corresponding 95% CI were calculated [10].

Our secondary outcomes of interest included reported causes of mortality and reported independent risk factors associated with early mortality in multivariate analysis. We reported these risk factor associations as hazard ratios or odds ratios as appropriate. Lastly, we assessed the quality of studies by evaluating the chance of ascertainment bias, which was calculated from two parameters: loss-to-follow-up reported and method for ascertainment of mortality reported. One point each was assigned for the above two parameters. Thus, the scoring was such that: 2 points indicated a low chance of ascertainment bias, 1 point indicated moderate chance of ascertainment bias and 0 points indicated high chance of ascertainment bias. We also assessed reporting of HIV-RNA plasma concentration, use of prophylaxis for opportunistic infections, adherence, control of confounding factors, and accounting for loss-to-follow-up. Confounder control in the included studies was considered to be adequate if multivariate analysis was performed (e.g., Cox proportional hazards or logistic regression to identify independent risk factors).

## Results

**Figure 1:**
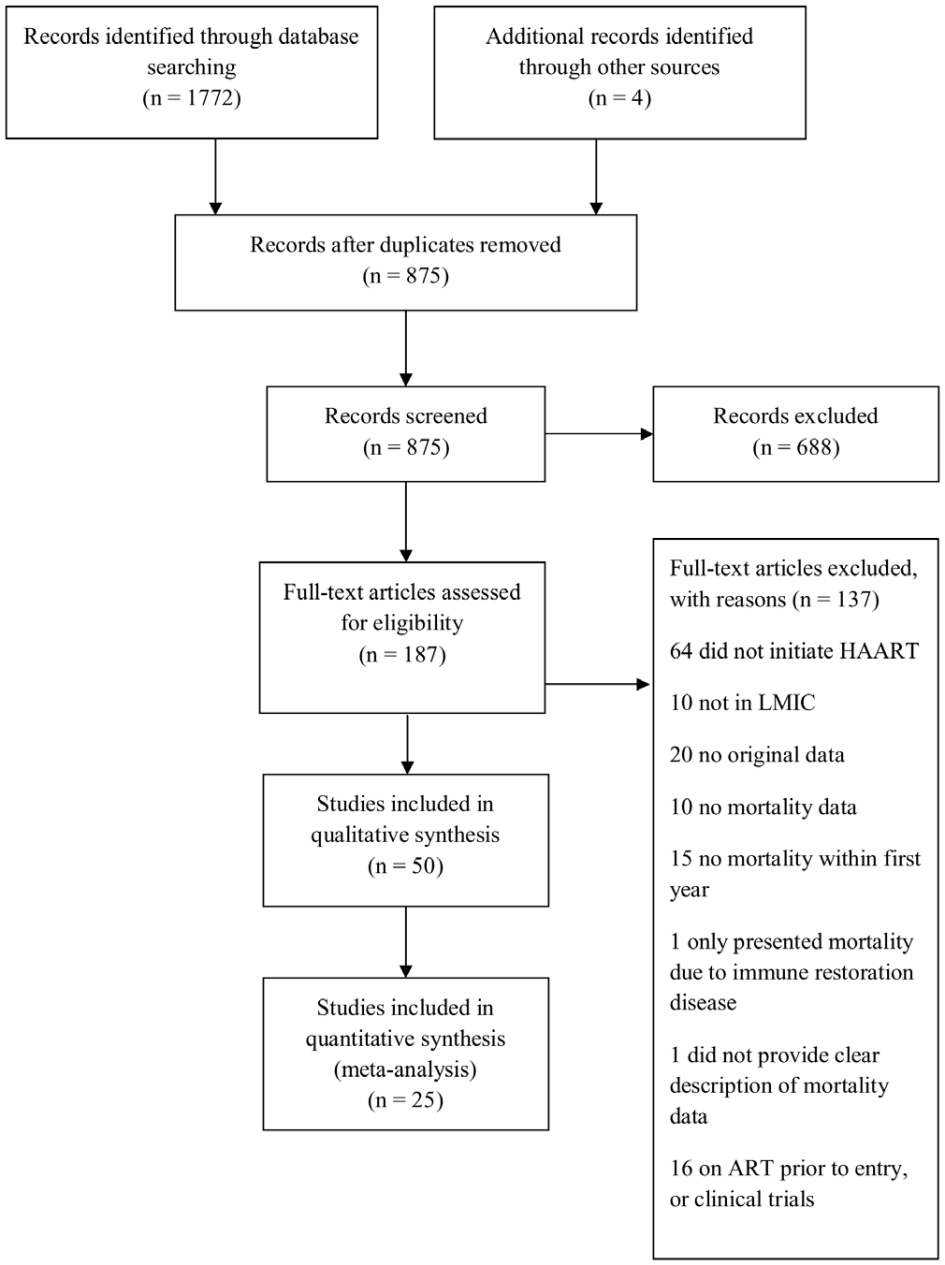
Study selection process and reasons for exclusion of studies. Flow chart constructed using the PRISMA guidelines (Moher D, Liberati A, Tetzlaff J, Altman DG, The PRISMA Group (2009). *P*referred *R*eporting *I*tems for *S*ystematic Reviews and *M*eta-*A*nalyses: The PRISMA Statement. PLoS Med 6(6): e1000097. doi:10.1371/journal.pmed1000097).

Of 875 articles, 187 studies received full text review, of which 50 met our inclusion criteria {**figure 1**}. Of 50 included studies, 38 (76%) were from SSA, five (10%) were multi-regional (Africa, Asia, South and Central America and the Caribbean), five (10%) were from Asia and two (4%) were from the Americas (South and Central Americas and the Caribbean) {**table 1**}. Most studies (n = 47) were published between the years 2005–2010, with the largest number of publications being in 2008 (n = 17).

**Table 1:** Characteristics of included studies^1^ and reported mortality and probability of survival at 12 months post-ART initiation by region.

Study	Year	Region^2^	Country	n (%F)	Loss to Follow-up, n (%)	Incidence of Death	Probability of Survival	Proportion of Death^3^
Alemu [26]	2010	SSA	Ethiopia	272 (57)	-	-	-	9.6%
Sanne [27]	2009	SSA	South Africa	7536 (67)	-	-	0.91–0.99^4^	-
Abaasa [28]	2008	SSA	Uganda	897 (75)	147 (16)	12.0/100py	-	18.0%
Banda [29]	2008	SSA	Malawi	81821 (61)	7753 (10)	-	0.61	-
Bisson [30]	2008	SSA	Botswana	410 (60)	22 (5)	-	0.83	-
Boulle [31]	2008	SSA	South Africa	12587 (70)	838 (7)	-	0.85	7.5%
Bussmann [32]	2008	SSA	Botswana	633 (60)	102 (16)	-	0.83	-
Hoffmann [33]	2008	SSA	South Africa	853 (2)	-	-	-	4.0%
Johannessen [34]	2008	SSA	Tanzania	320 (70)	31 (10)	-	0.71	-
Laurent [35]	2008	SSA	Cameroon	169 (67)	-	-	-	11.2%
MacPherson [36]	2008	SSA	South Africa	1353 (67)	35 (3)	7.5/100py	-	-
Marrrazi [37]	2008	SSA	Mozambique, Tanzania, and Malawi	3456 (60)	41 (1)	9.7/100py	-	7.5%
Mulenga [38]	2008	SSA	Zambia	25779 (60)	-	-	-	8·1%
Mzileni [39]	2008	SSA	South Africa	3073 (67)	434 (14)	-	-	6.5%
Toure [40]	2008	SSA	Cote d'Ivoire	10211 (70)	1385 (14)	-	0.77–0.94^5^	-
Yu [24]	2008	SSA	Malawi	2574 (-)	-	-	0.42, 0.82^6^	-
Karcher [41]	2007	SSA	Kenya	124 (81)	34 (27)	-	0.85	-
Makombe [42]	2007	SSA	Malawi	4580 (-)	511 (11)	-	0.87	12.7%
Makombe [43]	2007	SSA	Malawi	1022 (65)	40 (4)	-	0.81	-
Bekker [44]	2006	SSA	South Africa	1139(69)	33 (3)	-	-	5.0%-13.0%^7^
Etard [45]	2006	SSA	Senegal	404 (55)	16 (4)	-	0.88	11.6%
Ferradini [46]	2006	SSA	Malawi	1308 (64)	91 (7)	-	0.81	-
Lawn [47]	2006	SSA	South Africa	927 (72)	21 (2)	-	0.91	-
Stringer [4]	2006	SSA	Zambia	16198 (61)	3408 (21)	-	0.82	-
Zachariah [48]	2006	SSA	Malawi	1507 (66)	46 (3)	-	0·87	11.7%
Bourgeois [49]	2005	SSA	Cameroon	109 (66)	3 (3)	-	0.92	-
Wester [12]	2005	SSA	Botswana	153 (59)	- (8)	-	0.85	14.4%
Coetzee [50]	2004	SSA	South Africa	287 (70)	1 (0)	-	0.86	13.2%
Djomand [23]	2003	SSA	Cote d'Ivoire	490 (40)	-	-	0.84	-
Weidle [51]	2002	SSA	Uganda	476 (-)	114 (24)	-	0.74	-
Ruan [52] 8	2010	Asia	China	341 (46)	46 (14)	-	-	8.8%
Chasombat [53]	2009	Asia	Thailand	58008 (48)	5130 (9)	-	0.89	-
Morineau [54]	2009	Asia	Cambodia	549 (53)	not clear^9^	11.3/100py	-	-
Ferradini [55]	2007	Asia	Cambodia	416 (41)	7 (2)	-	0.87	-
Corey [56]	2007	Americas	Peru	564 (70)	-	-	0.97	-
Severe [3]	2005	Americas	Haiti	910 (55)	71 (8)	-	0.87	14.0%
O'Brien [57] 10	2010	Multi-regional	Africa & Asia	3757 (66)	413 (11)	-	0.89	9.0%
Tuboi [58]	2009	Multi-regional	Latin America, Caribbean	5152 (35)	297 (6)	-	0.92	-
Braitstein [5]	2006	Multi-regional	Africa, South America & Asia	4810 (51)	727 (15)	2.7/100py	0.94, 0.98^11^	-
Calmy [59]	2006	Multi-regional	Africa, South America & Asia	6861 (61)	328 (5)	-	0.90	-

**F** = female; **PY** = person years;

1 Only studies reported mortality measurements at 12 months post-ART initiation were listed. See {**table S2**} for summaries of papers not listed here [19], [60]–[68];

2 SSA: Sub-Saharan Africa; Americas: South & Central America, Caribbean; Multi-regional: includes SSA, Asia and the Americas;

3 Deaths expressed as percent of total population in the study;

4 Probability of survival was only presented by CD4 cell count level: CD4< = 50: 0.91, CD4 51–100: 0.97, CD4 101–200: 0.98, CD4>200: 0.99.

5 Probability of survival was only presented by CD4 cell count level: CD4< = 50: 0.77, CD4 51–100: 0.86, CD4 101–150: 0.91, CD4>150: 0.94.

6 Probability of survival was only presented by TB status: with TB: 0.42, without TB: 0.82.

7 Proportion was reported during each calendar year: 2002–'03: 13.0%, 2003–'04: 7.9%, 2004–'05: 5.0%;

8 Assumed death and loss-to-follow-up were 12 month data since only 12-month assessment was mentioned.

9 Loss to follow-up was reported but time was not given;

10 Supplement table 3 was used to obtain data for adults only;

11 Probabilities were reported in both active (0.94) and passive (0.98) follow-up groups.

### Base-line characteristics

The majority (48%) of studies were in an urban setting, 18% were in rural, 24% were in a mixed setting and 10% were unknown {**table S2**}. Cohort size ranged from 109–81,821 persons. The proportion of females ranged from 2%–81%. Thirty-four (68%) studies reported a median baseline CD4 cell count that ranged from 11–192 cells/mm^3^; median CD4 cell count was lowest from the Asian studies (11–41 cells/mm^3^). Fourteen (28%) studies reported median baseline log HIV-RNA concentrations that ranged from 4.6–5.7 log copies/ml. Median follow-up was reported by 42 (84%) studies and ranged from 3–55 months. Forty (80%) studies reported loss-to-follow-up, which overall ranged from 0.3%–27%. Lost-to-follow-up was highest in SSA.

### Mortality characteristics

**Figure 2:**
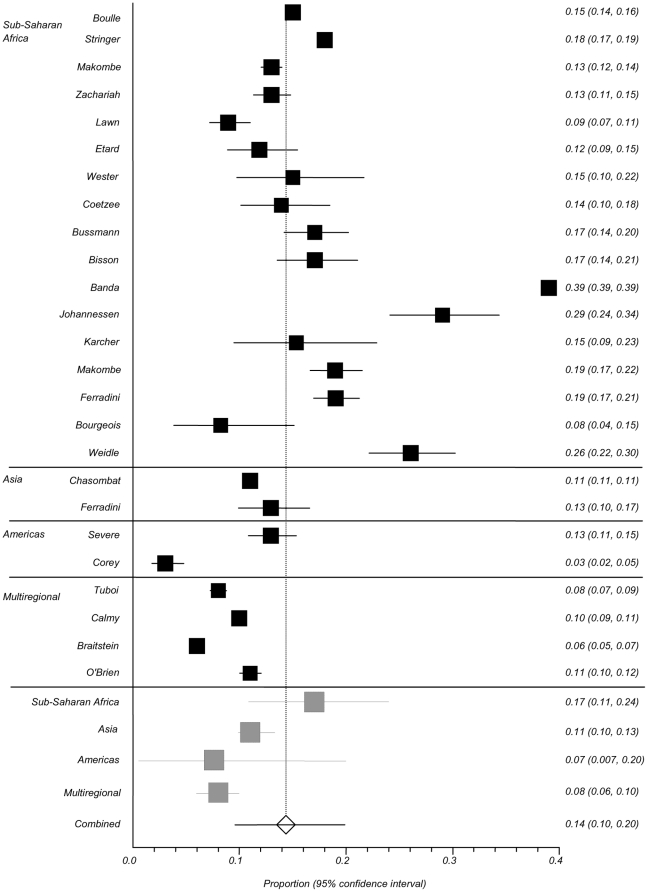
Forest plot of estimates of mortality at 12 months by individual studies and pooled by region. Pooled estimates are summary random effects estimates with 95% confidence intervals. Two studies were excluded as they did not provide appropriate data for pooling (Djomand [23] and Yu [24]). The summary pooled estimate is 0.14 (95% CI 0.10–0.20). Test for heterogeneity by region was as follows [7]: Sub-Saharan Africa Cochran Q = 7691, p-value<0.0001 and I^2^ = 99.84% (95% CI 99.8%–99.8%)- suggesting there is an evidence of heterogeneity among studies; Asia Cochran Q = 1.67; p = 0.20– suggesting non-heterogeneous studies (I^2^ cannot be estimated since only 2 studies); Americas Cochran Q = 51.54; p<0.0001 – suggesting there is evidence of heterogeneity (I^2^ cannot be estimated since only 2 studies) and Multiregional: Cochran Q = 90.7; p<0.0001 and I^2^ = 96.7% (95% CI 94.7%–98.7%) suggesting there is evidence of heterogeneity.

**Figure 3:**
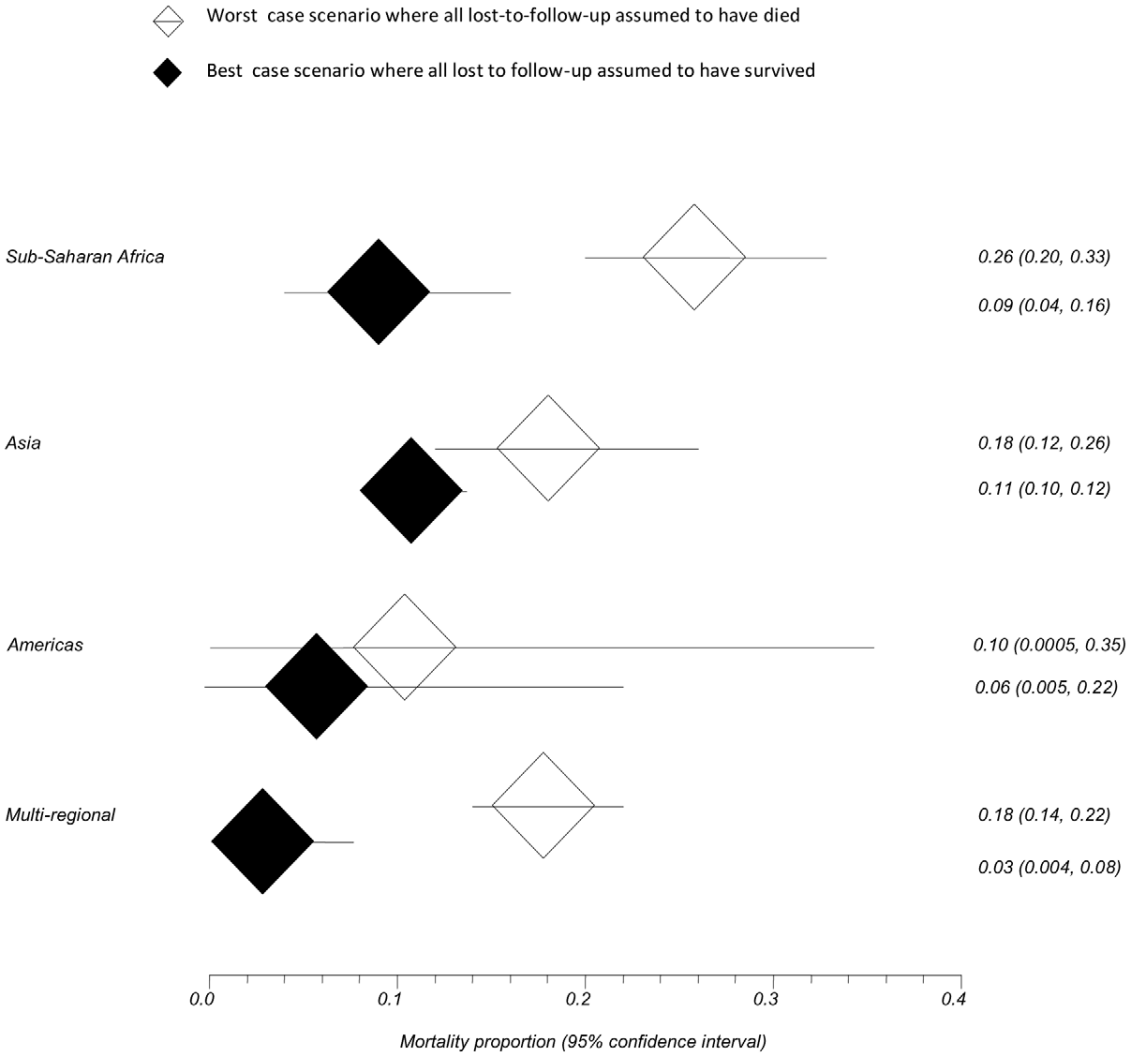
Sensitivity analyses for pooled regional estimates of mortality at 12 months by best case (all loss-to-follow-up assumed to have survived) and worst case (all lost-to-follow-up assumed to have died) scenarios.

Mortality at the end of follow-up varied by region and ranged from 2.6%–29.7%, with the lowest reported from the South American cohort of a multi-regional study and highest from SSA. Forty (80%) studies reported on 12 month post-ART initiation mortality however method of mortality reporting varied {**table 1**}. The overall pooled estimate of mortality at 12-months post-ART initiation was 0.14 (95% CI 0.10–0.20) but varied substantially by region. The highest mortality estimate was in SSA 0.17 (95% CI 0.11–0.24) followed by Asia 0.11 (95% CI 0.10–0.13); the Americas 0.07 (0.007–0.20); and multiregional 0.08 (95% CI 0.06–0.10) {**figure 2**}. A sensitivity analysis to assess the best case scenario where all loss-to-follow-up were assumed to have survived versus the worst case scenario where all loss-to-follow-up were assumed to have died is shown in **figure 3**. The majority of deaths were found to have occurred in the first three months after ART initiation {**table S2**}.

### Baseline risk factors associated with early mortality

Thirty-one (62%) studies [SSA, n = 24; Asia, n = 2; Americas, n = 1; Multi-region, n = 4] assessed independent risk factors associated with early mortality {**table 2**}. We did not report one of the 31studies due to insufficient data (strength of association of independent risk factors in terms of hazard or odds ratios were not reported) [12]. Factors assessed and reported to have independent association were: low baseline CD4 cell count (n = 23); advanced World Health Organization (WHO) clinical stage (n = 20); low body mass index (BMI) (n = 11); male sex (n = 9); anemia (n = 8); age (n = 5); weight (n = 4); ART adherence (n = 3); cotrimoxazole use (n = 2); total lymphocyte count (n = 2); high HIV RNA (n = 1); adherence to visits (n = 1); calendar year (n = 1); any education (n = 1); renal function (n = 1); Kaposi's sarcoma (n = 1); hospital type (n = 1); ART regimen (n = 1); tuberculosis (TB) clinic on site (n = 1); platelet count (n = 1); and fluconazole use (n = 1) {**table S3**}. When using standardized definitions across studies as shown in **table 2**, pre-ART CD4 cell count <50 cells/mm^3^, male sex, WHO clinical stage 4, low BMI<18.5, hemoglobin <8 g/dL, age >40 years, and pre-ART HIV RNA>5 log copies/ml carried the greatest risks for early mortality.

**Table 2:** Range of hazard and odds ratios of independent risk factors associated with early mortality by region.

Association	Low BMI	CD4(<50)	WHO Stage 4	Hb (<8 g/dL)	Age (≥40 years)	VL (>5 log copies/ml)	Male Sex
	n = 5	n = 13	n = 8	n = 4	n = 1	n = 1	n = 9
**Sub-Saharan Africa**							
Hazard ratio	1.93^1^–2.92	1.64–5.90	1.99–5.13^2^	3.10–9.20	-	-	1.52–2.22
Odds ratio	2.10^3^^,^ 4–6.00^3^^,^ 5	2.20–2.93	2.10^3^	2.10^3^	-	2.00^3^	-
**Asia**							
Hazard ratio	2.47^3^^,^ 6	2.43^3^	1.86^3^	-	1.24^3^	-	1.96^3^
**Americas**							
Hazard ratio	-	1.60^3^	-	-	-	-	-
**Muti-regional**							
Hazard ratio	-	1.27^7^–3.34^8^	3.86^3^	2.62^3^	-	-	1.75^3^

Factors included in the table are from the studies that reported risk factors having independent association with early mortality in multivariate analysis. We used most commonly used cutoff values to summarize findings across different studies, therefore factors found associated with early mortality using other definitions were not presented in this table. See {**table S3**} for complete details. **BMI** = Body Mass Index, all BMI values are in kg/m^2^ and <18.5 unless mentioned otherwise; **CD4** = CD4 cell count, all CD4 counts are <50 cells/mm^3^ unless mentioned otherwise; **WHO stage 4** = World Health Organization clinical stage 4; **Hb** = Hemoglobin, hemoglobin values are <8 gm/dL; **Age** is >40 years unless mentioned otherwise; **VL** = Viral Load;

1 BMI<18.5 vs BMI>25;

2 Using the real-case model in Alemu 2010 paper [26];

3 Reported by only one study;

4 For BMI in the range 17–18.4;

5 For BMI<15.9;

6 Hazard ratio 2.47 for BMI<17 vs BMI> = 18.5;

7 CD4 50 vs CD4 100;

8 CD4<25 vs CD4> = 50.

### Causes of mortality

Fourteen (28%) studies reported cause-specific mortality [SSA, n = 11; Asia, n = 1; Americas, n = 2] {**table 3**}. Most common causes of death reported were TB accounting for 5%–44% of deaths; advanced HIV 20%–37%; wasting 5%–53%; chronic diarrhea 10%–25%; Cryptococcal meningitis 3%–18%; and Kaposi's sarcoma 3%–12%. Five (36%) of 14 studies reported a specific method for ascertaining the cause of death, of which two used verbal autopsy along with patient records. Most of the studies reported the causes of deaths based on clinical diagnosis but not microbiological confirmation.

**Table 3:** Reported causes of death in included early mortality studies by region.

Study (Year)	Total Deaths, n (%)	Known Causes, n (%)	Causes of Death, n (%)							Methods to Ascertain Deaths^1^
			TB	Advanced HIV	Wasting	CM	KS	Chronic Diarrhea	Others	
**Sub-Saharan Africa**										
Bussmann [32] (2008)	120 (19.0)	-	8 (6.7)	41 (34.2)^2^	See footnote^2^	-	-	-	-	a, b^3^
Laurent [35] (2008)	19 (11.2)	19 (100.0)	1 (5.3)	6 (31.6)	1 (5.3)	-	-	-	Poor general health 6 (31.6); hepatic carcinoma 1 (5.3); hepatitis & pancreatitis 1 (5.3); persistent FUO 1 (5.3); malaria 1 (5.3); pulmonary infection 1 (5.3)	NS
MacPherson [36] (2008)	124 (9.1)	106 (85.5)	47 (44.3)	-	-	3 (2.8)	3 (2.8)	26 (24.5)	Carcinoma 4 (3.8); cerebral space occupying lesion 4 (3.8); septicemia 4 (3.8); hepatic failure 3 (2.8); obstetric 2 (1.9); bacterial meningitis 2 (1.9); congestive cardiac failure 2 (1.9); pneumonia 2 (1.9); HIV encephalopathy 1 (0.9); diabetic ketoacidosis 1 (0.9); renal failure 1 (0.9); upper GI bleed 1 (0.9)	NS
Marazzi [37] (2008)	260 (7.5)	122 (46.9)	23 (18.9)	-	-	-	-	-	Malaria 39 (32.0); anemia 35 (28.7)	NS
Mzileni [39] (2008)	205 (7.8)	204 (99.5)	42 (20.6)	76 (37.3)	-	18 (8.8)	11 (5.4)	25 (12.3)	Bacterial pneumonia/PCP 12 (5.9); lactic acidosis 12 (5.9); lymphoma 6 (2.9); hepatitis 6 (2.9); stroke 3 (1.5)	NS
Karcher [41] (2007)	15 (12.1)	11 (73.3)	4 (36.4)	-	1 (9.1)	2 (18.2)	1 (9.1)	-	PCP 2 (18.2); gastroenteritis 1 (9.1)	NS
Etard [45] (2006)	93 (23.0)	80 (86.0)	17 (21.3)	-	-	-	2 (2.5)	-	Neurological disorders 17 (21.3); septicemia 17 (21.3); gastro-intestinal infections 10 (12.5); respiratory 6 (7.5); hepatitis 5 (6.3); metabolic disorder 3 (3.8); other 3 (3.8)	a, b
Zachariah [48] (2006)	190 (12.6)	105 (55.3)	10 (9.5)	-	6 (5.7)	7 (6.7)	13 (12.4)	10 (9.5)	Oral Candidiasis 26 (24.8); esophageal Candidiasis 15 (14.3); severe bacterial pneumonia 12 (11.4); chronic fever 4 (3.8); PCP 2 (1.9)	a
Bourgeois [49] (2005)	9 (8.3)	9 (100.0)	1 (11.1)	2 (22.2)	1 (11.1)	-	-	-	Poor general health 2 (22.2); FUO 1 (11.1); hepatitis & pancreatitis 1 (11.1); hepatic carcinoma 1 (11.1)	NS
Wester [12] (2005)	24 (15.7)	20 (83.3)	4 (20.0)	4 (20.0)	2 (10.0)	1 (5.0)	2 (10.0)	-	Hepatotoxicity 2 (10.0); anemia 1(5.0); lymphoma 1(5.0); renal failure 1(5.0); suicide 1(5.0); traditional medicine toxicity 1(5.0)	c
Coetzee [50] (2004)	38 (13.2)	38 (100.0)	3 (7.9)	20 (25.6)	-	-	3 (7.9)^4^	-	Treatment discontinuation/poor adherence 6 (15.8); not attributed to HIV 2 (5.3); CMV colitis1 (2.6)	NS
**Asia**										
Chasombat [53] 5 (2009)	7637 (13.2)	5616 (73.5)	-	-	-	-	-	-	-	NS
**Americas**										
Corey [56] (2007)	16 (2.8)	13 (81.3)	2 (15.4)	-	4 (30.8)	1 (7.7)	1 (7.7)	-	Pulmonary insufficiency 2 (15.4); lymphoma 1 (7.7); CMV 1 (7.7); sepsis 1 (7.7)	a
Severe [3] (2005)	127 (14.0)	104 (81.9)	20 (19.2)	-	55 (52.9)	-	-	-	Bacterial pneumonia 6 (5.8); Toxoplasmosis 5 (4.8); malignancy 4 (3.8); Cryptosporidiosis 4 (3.8); sepsis 4 (3.8); congestive heart failure 3 (2.9); trauma 3 (2.9)	NS

**TB** = Tuberculosis; **CM** = Cryptococcal Meningitis; **KS** = Kaposi's Sarcoma; **FUO** = Fever of Unknown Origin; **PCP** = Pneumocystis Carinii Pneumonia; **CMV** = Cytomegalovirus;

1 a: Clinical record; b: Verbal autopsy; c: Active ascertainment but unspecified; NS: not specified;

2 Advanced HIV and wasting were reported together;

3 Information available in 71 deaths;

4 Probable KS;

5 Deaths were only recorded as due to AIDS or unknown. All deaths with known causes (n = 5616) were due to AIDS.

### Quality of studies

**Figure 4:**
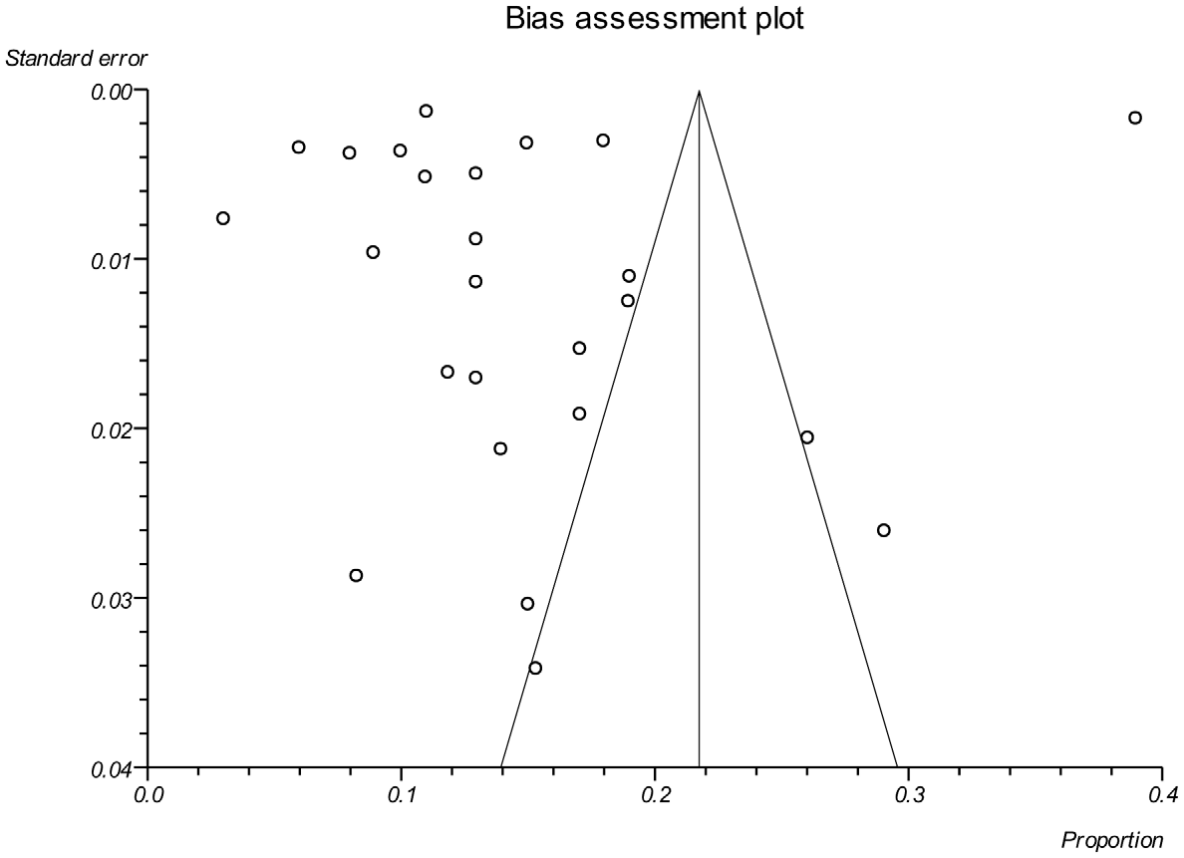
Funnel plot. The funnel plot assesses the hypothesis that the relationship between probability of death and study size, measured by standard error, is independent. This was tested using a Kendall's tau, which was estimated to be 0.4 with p-value = 0.0046, suggesting there is evidence of asymmetry. Although the presence of publication bias is a common explanation to an asymmetric funnel plot, data presented here are observational data without any intervention so the funnel plot asymmetry could also be due to heterogeneity in the data [25].

Most studies (92%) had low-to-moderate risk of ascertainment bias. However, only a small proportion disclosed prophylaxis for opportunistic infection (24%), or mentioned adherence (38%) {**table S4**}. There was evidence of heterogeneity or reporting bias as assessed by a funnel plot (Kendall's tau was 0.4 with p-value = 0.0046) {**figure 4**}.

## Discussion

In our study, we carefully reviewed and summarized the magnitude of early deaths occurring within the first 12 months post-ART initiation in different LMIC regions. Such data are needed by healthcare providers, policy makers, program funders alike to determine resource allocation and to optimize care and treatment strategies for HIV in LMIC. More than three-quarters of our included studies were from SSA. While this appears to reiterate that SSA continues to bear a large portion of global HIV burden, it also points to the relative lack of published data on early mortality from LMIC outside of SSA.

An overall estimate of mortality 12 months post-ART initiation was 14% with the highest probability being reported from SSA at 17%, followed by Asia at 11%, and the Americas at 7%. These differences could represent differences in factors such incidence of opportunistic infections, nutrition levels, socioeconomic levels and disease stage of study participants. However, it is also likely that some of the difference may be due to varying differences in economic and health infrastructure across regions. Consistent with this, Lawn *et al* have demonstrated an inverse ecologic relationship between growth domestic product (GDP) per capita and mortality proportion for 21 cohorts assessed [13]. However the specific reasons for such differences between regions could not be specifically teased out in our systematic review based on the available reported data.

Among the studies that reported on deaths within the first year of ART initiation, we observed that most deaths occurred within the first 3 months of ART initiation. This reflects the advanced stage of HIV disease of many participants in whom ART was initiated and is evidenced by the finding that more than 50% of included studies reported a median baseline CD4 cell count at or below 150 cells/mm^3^ and that the vast majority of patients had advanced HIV disease stage as ascertained by WHO clinical staging. It is clear from several published studies that starting ART at a higher CD4 cell count will reduce morbidity and avert the high mortality rates seen among patients with advanced HIV disease [14], [15]. For this reason, there has been a call for more aggressive testing and treating of HIV-infected persons upon identification of their HIV status irrespective of their CD4 cell count or disease stage as several decision models suggest that this is an optimal strategy to reduce HIV-associated morbidity and mortality in LMIC [16]. However, the cost-effectiveness and implementation of such a strategy remain unknown. Other strategies to reduce early mortality are likely to include better screening, prevention and management of opportunistic infections such as TB, better pre-ART care, and retention of patients in HIV programs. However, where to best focus limited financial resources needs further evaluation. Irrespective of what strategies are adopted, late presenters to care (HIV-infected patients with CD4 cell counts less than 200 cells/mm^3^) will remain an ongoing reality. Therefore, understanding the risks of death and assessing strategies to deal with early mortality in this vulnerable population remain critical.

Only one-third of included studies reported causes of death and the majority of these were from SSA. TB and wasting appeared to be the most common causes of early mortality. It is unclear as to what proportion of wasting in patients could be attributed to TB or other opportunistic infections, but it is likely that TB has some substantial contribution as an autopsy study in South Africa identified TB as the most common cause of death in patients receiving ART [17]. Approaches to reducing TB burden as a contributor to early mortality are needed and will likely include scaling up of TB prevention among HIV- positive adults seeking to start ART, using novel rapid TB detection methods such as Cepheid Gene Xpert MTB, and assessing the role of pre-emptive TB therapy along with starting ART in those at highest risk of early mortality, such as those with very advanced HIV disease. The latter strategy is being assessed by the AIDS Clinical Trials Group 5274 REMEMBER trial (NCT 1380080).

Other independent risk factors for early mortality included older age and male sex. Interestingly, older age has been associated with later presentation, diagnostic delays, as well as with immune senescence and poorer CD4 immune reconstitution, which may in part explain the findings [18], [19].The observation that males were in general more likely to die early in the course of therapy than females may be because of discrepancies in healthcare seeking behavior between the sexes or poorer adherence in men or due to biological differences in ART response, but these differences need to be further examined as prior studies have found mixed evidence for sex differences in HIV disease progression, adherence and HIV treatment outcomes [20]–[22].

While several studies have alluded to the underlying association between early mortality and poor nutrition, few studies have directly assessed this association. Most studies that have reported on nutrition have used BMI, body weight or hemoglobin levels as proxy markers of nutrition. While these are frequently used correlates of nutrition, their utility is confounded by the existence of co-morbidities in patients with HIV that may influence the body weight, BMI or hemoglobin levels. For example, anemia can be due to non-nutritional causes such as anemia of chronic disease due to HIV infection and likely represents a large proportion of the patients who were defined as having anemia in the included studies. However, iron deficiency anemia or anemia due to other micronutrient deficiencies were not ascertained in any of the studies, therefore the role of malnutrition-induced anemia contributing to early mortality remains inadequately assessed.

Our study had a few limitations. Like any systematic review, there is the possibility of incomplete retrieval or abstraction of data; however, we used three independent reviewers to try and best address this. Furthermore, we did not obtain raw data from study investigators for pooled estimation of mortality; however, we used only those studies from which appropriate Kaplan-Meier data could be extracted using published methods. There was also substantial heterogeneity and/or reporting bias among the studies, so the pooled estimates have to be understood in that context. Despite this heterogeneity, we felt it was important to pool the existing data to estimate mortality probability at 12 months as these data provide a more robust estimate than any single study alone. Furthermore, we performed a sensitivity analysis to assess best and worst case scenarios for mortality estimates based on assumptions made for those who were loss-to-follow-up. In addition, we could not pool risk factors or the reported causes of death such as low CD4 cell count or TB, respectively, as there were insufficient data included in the published studies to perform this.

In conclusion, more studies are needed to report on factors that affect early mortality in LMICs outside of SSA- in particular, high burden regions like South and Southeast Asia and Latin America. Future studies also need to focus on rural populations, more specific nutritional assessment and improved ascertainment of causes of death. Lastly, assessments of interventions directed at late presenters with advanced HIV are needed, as even with novel approaches such as HIV test-and-treat strategies, there will likely continue to be HIV-infected patients who present with advanced disease and remain at high risk for early mortality despite ART initiation.

## Supporting Information

Table S1 **Exact search terms.** The final search strategy was the combination of search terms 1, 2 and 3.
(DOC)

Table S2 **Characteristics and mortality data from all studies of patients initiating ART in low- and middle-income countries grouped by region and by method of death reporting.** **F** = female; **IQR** = Inter quartile Range; **CD4** cell count = cells/mm^3^; **SD** = Standard deviation; **PY** = Person Years; Two studies had a mix of active and passive follow-up among their clinics. However, studies defined lost to follow-up in various ways and used different methods for tracing participants. **§** = Two studies used passive reporting and 11 active tracing, latter of which included home visits/community visits (n = 9), phone calls (n = 4) and letters (n = 2); others did not specify; **#** = Multivariable analysis of risk factors of death performed.
(DOCX)

Table S3 **Studies assessing independent risk factors for early mortality organized by measure of association and by region.** **^Ω^**only those measures of association reaching statistical significance in multivariate analysis are included; **BMI** = Body Mass Index. All BMI values are baseline and in kg/m^2^ unless mentioned otherwise; **CD4** = CD4 cell count. All CD4 counts are baseline and in cells/mm^3^ unless mentioned otherwise; **Clinical staging** = World Health Organization (WHO) Clinical staging unless mentioned otherwise; **Hb** = Hemoglobin, Hemoglobin values are baseline and in gm/dL unless mentioned otherwise; **Age** is in years unless mentioned otherwise; **Viral Load** = HIV–1 RNA plasma concentration, Viral load is in log copies/ml unless mentioned otherwise; **TLC** = Total leukocyte count, TLC is cells/mL unless mentioned otherwise; **CTX** site = Cotrimoxazole treatment site; **TB** = Tuberculosis; **KS** = Kaposi's Sarcoma; **ART** = Anti-retroviral therapy; **HAART** = Highly active antiretroviral therapy; **F** = Female; **M** = Male.
(DOCX)

Table S4 **Qualitative assessment of included studies.** **^1^**Ascertainment bias was calculated from two parameters, a) Loss to follow–up mentioned and b) Active method for ascertainment of mortality. If loss to follow–up was mentioned 1 point was assigned and if there was an active method for ascertainment of mortality then 1 point was assigned. The scoring system was thus: **2 points**- Low chance of ascertainment bias; **1 point**- Moderate chance of ascertainment bias; **0 points**– High chance of ascertainment bias; **^2^**Cotrimoxazole/Tuberculosis prophylaxis; **^3^**Adherence was evaluated in various methods such as assigning a care taker for the patient, prescribing medication for a standard duration and asking the patients to come with the completed kit, counseling the patients and their care takers at the initiation of antiretroviral therapy about the importance of being adherent to medication;^4^Confounder control was said to be adequate if multivariable analysis was performed; ^5^Losses to follow–up was defined in various forms by each study and was accounted by different means such as making phone calls, home visits, attendance from patient registers etc; ^6^Adherence counseling was done to patients/patient guardian at the time of initiation of ART but adherence was never measured during the follow–up; ^7^211(3.8%) were lost to follow–up after 1^st^ART visit while 880(16%) were lost to follow–up later on.
(DOCX)
