# Supplementary material for: Early Mortality in Adults Initiating Antiretroviral Therapy (ART) in Low- and Middle-Income Countries (LMIC): A Systematic Review and Meta-Analysis
Source: PLoS One. 2011 Dec 29;6(12):e28691. doi: 10.1371/journal.pone.0028691 (PMC3248405; doi:10.1371/journal.pone.0028691)
Supplement: Table S1 — Exact search terms. The final search strategy was the combination of search terms 1, 2 and 3. (DOC) [file pone.0028691.s001.doc]

| Search Terms 1 | developing countries (mh) OR africa (mh) OR south america (mh) OR asia (mh) OR developing country (tiab) OR developing countries (tiab) OR africa (tiab) OR south america (tiab) Or asia (tiab) |
| --- | --- |
| Search Terms 2 | mortality (sh) OR mortality (mh) OR death (tiab) OR deaths (tiab) OR mortality (tiab) |
| Search Terms 3 | antiretroviral therapy, highly active (mh) OR haart (tiab) Or anti-retrovirals (tiab) OR antiretrovirals (tiab) OR antiretroviral (tiab) |

**Legend:** The final search strategy was the combination of search terms 1, 2 and 3.
