# Supplementary material for: Early Mortality in Adults Initiating Antiretroviral Therapy (ART) in Low- and Middle-Income Countries (LMIC): A Systematic Review and Meta-Analysis
Source: PLoS One. 2011 Dec 29;6(12):e28691. doi: 10.1371/journal.pone.0028691 (PMC3248405; doi:10.1371/journal.pone.0028691)
Supplement: Table S2 — Characteristics and mortality data from all studies of patients initiating ART in low- and middle-income countries grouped by region and by method of death reporting. F = female; IQR = Inter quartile Range; CD4 cell count = cells/mm3; SD = Standard deviation; PY = Person Years; Two studies had a mix of active and passive follow-up among their clinics. However, studies defined lost to follow-up in various ways and used different methods for tracing participants. § = Two studies used passive reporting and 11 active tracing, latter of which included home visits/community visits (n = 9), phone calls (n = 4) and letters (n = 2); others did not specify; # = Multivariable analysis of risk factors of death performed. (DOCX) [file pone.0028691.s002.docx]

|  | | | | | | | | | | | | | | | | |
| --- | --- | --- | --- | --- | --- | --- | --- | --- | --- | --- | --- | --- | --- | --- | --- | --- |
| **Study** | **Year** | **Country** | **n (%F)** | **Setting** | | **Median Baseline CD4 Cell Count**  **(IQR)** | **Median Baseline log HIV-1 RNA**  **(IQR)** | **Median Follow-up Months**  **(IQR)** | **Number of Deaths at End of Follow-up,**  **n (%)** | **Lost to Follow-up,**  **n (%)^§^** | **Temporal Distribution of Cumulative Deaths in First12 Months** | | | | **Multi-variate Analysis^#^** | **Causes of Death Specified** |
|  |  |  |  |  | |  |  |  |  |  | **1 month** | **3 months** | **6 months** | **12 months** |  |  |
| **Grouped by absolute no. of death / proportion of deaths** | | | | | |  |  |  |  |  |  |  |  |  |  |  |
| **sub-Saharan Africa** | | |  | |  |  |  |  |  |  |  |  |  |  |  |  |
| Alemu | 2010 | Ethiopia | 272(57) | | - | 103(range 1-423) | - | - | 28(10.3) | 48(18.0) | - | - | - | 93% | Yes | No |
| Rougement | 2009 | Cameroon | 312(63)^1^ | | - | 104(50-177) | - | 6 | 28(9.0) | 51(17.0) | - | - | 100% | - | No^2^ | No |
| De Beaudrap | 2008 | Senegal | 404(55) | | Mixed | 128(54-217) | 5·2(4.7-5.6) | 46 | 93(23.0) | 4(1.0) | - | - | 33% | - | Yes | No |
| Boulle | 2008 | South Africa | 12587(70) | | Rural | < 50: 51.3 - 21.5 % from 2001- 05 | - | 48 | 1522(12.1) | 838(6.6) | - | - | 36% | 62% | No | No |
| Hoffmann | 2008 | South Africa | 853(2) | | - | 186(101-282) | 4.7(4.3-5.2) | 12 | 34(4.0) | - | - | - | - | 100% | No | No |
| Laurent | 2008 | Cameroon | 169(67) | | Urban | 152(67-223)^3^  117(68-188)^4^ | 5.3(4.8-5.6)^3^  5.2(4.7-5.5)^4^ | 22^3^  24^4^ | 19(11.2) | - | - | - | 53% | 100% | Yes | Yes |
| Marazzi | 2008 | Mozambique, Tanzania, and Malawi | 3456(60) | | Urban | 166(80-257) | 4.7(4.1-5.2) | 12 | 260(7.5) | 41(1.2) | - | 53% | 69% | 100% | Yes | Yes |
| Mulenga | 2008 | Lusaka, Zambia | 25779(60) | | Mixed | Mean 157  (SD: 114)^5^/Mean 132 (SD 106)^6^ | - | 40 | 2314(9.0) | - | - | 59% | 77% | 91% | Yes | No |
| Mzileni | 2008 | South Africa | 3073(67) | | Urban | - | - | 30 | 205(6.7) | 434(14.1) | - | - | - | 95% | No | No |
| Nakanjako | 2008 | Uganda | 559 (69) | | Urban | 98(21-163) | - | 22(3-22) | - | 19(3.4) | - | - | 68 | - | No | No |
| Lowrance | 2007 | Malawi | 1052(62) | | Mixed | <200: 13.3% | - | 6 | 130(12.4)^7^ | - | - | - | 100% | - | Yes | No |
| Makombe | 2007 | Malawi | 4580(-) | | Rural | - | - | 12 | 580(12.7)^8^ | 511 (11.2)^9^ | - | - | - | 100% | No | No |
| Bekker | 2006 | South Africa | 1139(69) | | Urban | 82(42-139)^10^  89(49-149)^11^  110(55-172)^12^ | 5.0(4.65.3)^10^ 4.9(4.6-5.2)^11^  4·7(4.3-5.1)^12^ | 12 | 78(6.8) | 33(2.8) | - | - | - | 2003-27%  2004-28%  2005-45% | No | No |
| Etard | 2006 | Senegal | 404(55) | | Urban | 128(54-217) | 5.2(4.7-5.6) | 46(32-57) | 93(23.0) | 16(3.9) | - | - | - | 51% | Yes | Yes |
| Jerene | 2006 | Ethiopia | 152(43) | | Urban | - | - | 55(29-71) | 24(15.8) | - | 33% | - | 75% | - | Yes | No |
| Libamba | 2006 | Malawi | 13183(60) | | Mixed | - | - | 12 | 1026(7.8) | 1039 (8.0) | - | - | - | - | No | No |
| Stringer | 2006 | Zambia | 16198(61) | | Urban | Mean 143 (SD: 123) | - | 7(3-11) | 1142(7.1) | 3408(21.0) | - | 69% | - | 98% | Yes | No |
| Zachariah | 2006 | Malawi | 1507(66) | | Rural | 123(58-206) | - | 24 | 190(12.6) | 46(3.0) | - | 61% | 79% | 93% | Yes | Yes |
| Wester | 2005 | Botswana | 153(59) | | Urban | 96(33-165) | 5.6(5.2-5.9) | 14 | 24(15.7) | -(8·4) | - | 29% | 67% | 92% | Yes^13^ | Yes |
| Coetzee | 2004 | South Africa | 287(70) | | Urban | 43(13-94) | 5.2(SD: 0.68) | 14(10-18) | 38(13.2) | 1(0.3) | - | 71% | 87% | 100% | Yes | Yes |
| **Asia** |  |  |  | |  |  |  |  |  |  |  |  |  |  |  |  |
| Ruan | 2010 | China | 341(46) | | Rural | 0-99: 35.8%  100-199: 23.7%  200-349: 34.9%  >=350: 5.6% | - | 12.1 | 30(8.8) | 46(13.5)^14^ | - | - | - | 100% | No | No |
|  |  |  |  | |  |  |  |  |  |  |  |  |  |  |  |  |

| **Study** | **Year** | **Country** | **n (%F)** | **Setting** | | **Median Baseline CD4 cell count**  **(IQR)** | **Median Baseline log HIV-1 RNA**  **(IQR)** | **Median Follow-up Months**  **(IQR)** | | **Number of Deaths at End of Follow-up,**  **n (%)** | **Lost to Follow-up,**  **n (%)^§^** | **Temporal Distribution of Cumulative Deaths in First12 Months** | | | | **Multi-variate Analysis^#^** | **Causes of Death Specified** |
| --- | --- | --- | --- | --- | --- | --- | --- | --- | --- | --- | --- | --- | --- | --- | --- | --- | --- |
|  |  |  |  |  | |  |  | |  |  |  | **1 month** | **3 months** | **6 months** | **12 months** |  |  |
| Madec | 2007 | Cambodia | 1735(42) | | Urban | 20(6-78) | - | | 13(5-21) | 186(10.7) | 37(2.1) | - | 55% | - | - | Yes | No |
| **Americas** |  |  |  | |  |  |  | |  |  |  |  |  |  |  |  |  |
| Severe | 2005 | Haiti | 910(55) | | Urban | 131(55-211) | - | | 13 | 127(14.0) | 71(7.8) | - | - | 79% | 100% | Yes | Yes |
| **Multi-Regional** |  |  |  | |  |  |  | |  |  |  |  |  |  |  |  |  |
| Brinkhof | 2008 | Africa, South America & Asia | 5491(46) | | Mixed | 105(35-210) | - | | 6 | 141(2.6) | 880 (16.0) | - | - | 100% | - | Yes | No |
| **Grouped by probability of survival** | | | | |  |  |  | |  |  |  |  |  |  |  |  |  |
| **sub-Saharan Africa** | | |  | |  |  |  | |  |  |  |  |  |  |  |  |  |
| Alibhai | 2010 | Uganda | 385(58) | | Rural | M 120(58-183)  F 147(82-206) | - | | - | 42(10.9) | - | M:0.98  F:0.97 | M:0.91  F:0.94 | M:0.86  F:0.91 | - | Yes | No |
| Palombi | 2009 | Mozambique, Malawi and Guinea-Conakry | 3749(62) | | Mixed | 192(90-293) | 4.6(3.9-5.1) | | 36 | 393(10.5) | - | - | - | 0·94 | - | Yes | No |
| Sanne | 2009 | South Africa | 7536(67) | | Urban | 87(31-158) | - | | 20.3 | 385(5.1) | 1234(16.4) | CD4<=50: 0.99  CD4 51-100: 0.99  CD4 101-200: 0.99  CD4 >200:0.99 | CD4<=50: 0.96  CD4 51-100: 0.98  CD4 101-200: 0.99  CD4 >200:0.99 | CD4<=50: 0.91  CD4 51-100: 0.98  CD4 101-200: 0.99  CD4 >200:0.99 | CD4<=50: 0.91  CD4 51-100: 0.97  CD4 101-200: 0.98  CD4 >200: 0.99 | No | No |
| Banda | 2008 | Malawi | 81821(61)^15^ | | - | - | - | | 596PY | 9327(11.4) | 7753(9.5) | - | - | 0.74^16^ | 0.61^16^ | No | No |
| Bisson | 2008 | Botswana | 410(60) | | Urban | 81(31-145) | 5.7(5.1-5.9) | | 11(9-12) | 68(16.6) | 22(5.4) | - | - | - | 0.83 | Yes | No |
| Bussman | 2008 | Botswana | 633(60) | | Urban | 67(28-127) | 5.6(5.2-5.9) | | 42(8-57) | 120(19.0) | 102 (16.1) | - | - | 0.88 | 0.83 | No | Yes |
| Johannessen | 2008 | Tanzania | 320(70) | | Rural | - | - | | 11(3-20) | 95(29.7) | 31(9.7) | - | 0.81 | - | 0.71 | Yes | No |
| Toure | 2008 | Cote d’Ivoire | 10211(70) | | Urban | 123(47-207) | - | | 8(3-16) | 1140(11.2) | 1385 (14.0) | - | - | CD4<=50: 0.81  CD4 51-100: 0.89  CD4 101-150: 0.92  CD4 >150: 0.96 | CD4<=50: 0.77  CD4 51-100: 0.86  CD4 101-150: 0.91  CD4 >150: 0.94 | Yes | No |
| Yu | 2008 | Malawi | 2574^17^ | | Urban | - | - | | - | - | - | - | - | 0.86^18^; 0.48^19^ | 0.82^18^; 0.42^19^ | No | No |
| Karcher | 2007 | Kenya | 124(81) | | Rural | 189(15-536) | - | | 9(4-12) | 15(7.9) | 34 (27.0) | - | - | - | 0.85 | Yes | Yes |
| Makombe | 2007 | Malawi | 1022(65) | | Mixed | - | - | | 6&12 | 119(11.6) | 40(3.9) | - | - | 0.85 | 0.81 | No | No |
| Ferradini | 2006 | Malawi | 1308(64) | | Rural | 112(59-176) | - | | 8(6-13) | 243(18.6) | 91(7.0) | - | - | - | 0.81 | Yes | No |
| Bourgeois | 2005 | Cameroon | 109(66) | | Urban | 150(61-223) | 5.4(4.8-5.6) | | 16(11-23) | 9(8.3) | 3(2.8) | - | - | - | 0.92 | No | Yes |
| Djomand | 2003 | Cote d’Ivoire | 490(40) | | Urban | <50: 37% ; 50-199: 32%; 200-349:16%; 350-499: 6% ; >500: 4% | <4·0 : 7%  4·0-5·0 : 13%  >5·0 : 68% | | 6 | - | - | - | - | - | 0.84 | Yes | No |
| Weidle | 2002 | Uganda | 476^20^ | | Urban | 73(15-187) | 5.3(4.6-5.8) | | 3 | 74 | 114 (24.0) | **-** | - | 0.82 | 0.74 | Yes | No |
| **Study** | **Year** | **Country** | **n (%F)** | **Setting** | | **Median Baseline CD4 cell count**  **(IQR)** | **Median Baseline log HIV-1 RNA**  **(IQR)** | **Median Follow-up Months**  **(IQR)** | | **Number of Deaths at End of Follow-up,**  **n (%)** | **Lost to Follow-up,**  **n (%)^§^** | **Temporal Distribution of Cumulative Deaths in First12 months** | | | | **Multi-variate Analysis^#^** | **Causes of Death Specified** |
|  |  |  |  |  | |  |  | |  |  |  | **1 month** | **3 months** | **6 months** | **12 months** |  |  |
| **Asia** |  |  |  | |  |  |  | |  |  |  |  |  |  |  |  |  |
| Chasombat | 2009 | Thailand | 58008(48) | | Mixed | 41(13-113) | - | | 18(1-28) | 7637(13.2) | 5130(8.8) | **-** | - | - | 0.89 | Yes | Yes |
| Ferradini | 2007 | Cambodia | 416(41) | | Urban | 11(3-60) | - | | 24 | 53(12.7) | 7(1·7) | **-** | - | - | 0·87 | No | No |
| **Americas** |  |  |  | |  |  |  | |  |  |  |  |  |  |  |  |  |
| Corey | 2007 | Peru | 564(70) | | Urban | Mean 91(SD: not available) | - | | 35(range, 3days-7years) | 16(2.8) | - | **-** | - | - | 0.97 | No | No |
| **Multi - Regional** |  |  |  | |  |  |  | |  |  |  |  |  |  |  |  |  |
| O’Brien | 2010 | Africa & Asia^21^ | 3757(66)^22^ | | Mixed^23^ | 139(63-211) | - | | - | -(9) | -(11) | **-** | - | 0.92 | 0.89 | No | No |
| Tuboi | 2009 | Latin America, Caribbean | 5152(35) | | Mixed | 107(39-201) | 5.0(4.6-5.4) | | - | 399(7.7)^24^ | 297(5.8) | **-** | 0.95 | 0.93 | 0.92 | Yes | No |
| Calmy | 2006 | Africa, South America & Asia | 6861(61) | | Mixed | 89(33-158) | - | | 4(2-7) | 413(6.0) | 328 (4.8) | **-** | - | 0.93 | 0.90 | Yes | No |
| **Grouped by incidence / 100 person years** | | |  | |  |  |  | |  |  |  |  |  |  |  |  |  |
| **sub-Saharan Africa** |  |  |  | |  |  |  | |  |  |  |  |  |  |  |  |  |
| Sanne | 2009 | South Africa | 7536(67) | | Urban | 87(31-158) | - | | 20.3 | 385(5.1) | 1234(16.4) | - | 8.4 | 6.8 | - | No | No |
| Abaasa | 2008 | Uganda | 897(75) | | Urban | >50: 22%  <50: 78% | - | | - | 164(18.3) | 147(16.4) | **-** | - | - | 12.0^25^ | Yes | No |
| MacPherson | 2008 | South Africa | 1353(67) | | Rural | 93(37-148) | - | | 24 | 124(9.2) | 35(2.6) | **-** | - | 19.5 | 7.5 | Yes | Yes |
| Lawn | 2006 | South Africa | 927(72) | | Urban | 100(47-160) | 4.8(4.4-5.2) | | 7 | 65(7.0) | 21(2.3) | **-** | - | - | 1.3 | Yes | No |
| **Asia** |  |  |  | |  |  |  | |  |  |  |  |  |  |  |  |  |
| Morineau | 2009 | Cambodia | 549(53) | | - | 35 M  100 F | -- | | 13.2(6-24) | 37(6.7) | 22(4.0) | - | 20.1 | - | 11.3 | No^26^ | No |
| **Multi-Regional** |  |  |  | |  |  |  | |  |  |  |  |  |  |  |  |  |
| Braitstein | 2006 | Africa, South America & Asia | 4810(51) | | Mixed | 108(37-.210) | - | | 2236 PY | 165(3.4)^24,27^ | 727(15·1) | 14.7 | 5.1 | 5.1 | 2.7^28^ | Yes | No |

**Legend & Footnotes: F**= female; **IQR**= Inter quartile Range; **CD4** cell count= cells/mm^3^; **SD**=Standard deviation; **PY**= Person Years; Two studies had a mix of active and passive follow-up among their clinics. However, studies defined lost to follow-up in various ways and used different methods for tracing participants. **§** = Two studies used passive reporting and 11 active tracing, latter of which included home visits/community visits (n= 9), phone calls (n= 4) and letters (n= 2); others did not specify; **#**= Multivariable analysis of risk factors of death performed;

^1^3% of gender information was missing; ^2^No multivariate regression but univariate association was done;**^3^**InZDV sub-cohort;**^4^**In d4T sub-cohort**;^5^**Those with normal creatinine clearance;^6^Those with decreased creatinine clearance;^7^Fifty seven (10%) deaths in ART patients at cotrimoxazole treatment sites & seventy three (15%) deaths in ART patients at non-cotrimoxazole treatmentsites;**^8^**Forty four deaths (11%) in tuberculosis affected group and 536 (12%) in non-tuberculosis group;^9^Thirty one (8%) deaths in tuberculosis affected group and 479 (12%) in non-tuberculosis group;^10^From 2002 – 03;^11^From 2003–04;^12^From 2004 –05;^13^Multivariate analysis was done but insufficient data; ^14^Assume death and loss to follow up data was at 12 month since only first month and 12 month interviews were mentioned;^15^Includes non-army personnel (81274) and army personnel(547);^16^Survival probabilities of the population who accessed public section ART facilities;^17^Excluding359 (12%) children, and sex distribution was not available among adults;^18^Survival probability of those who did not develop TB (Children and adults included);^19^Survival probability of those who developed TB (Children and adults included); ^20^Data not separated and includes 449 adults (>13years), 27children (<13years);^21^Only 1 out of 12 programs was in India, but it consisted 21.9% of the total sample size; ^22^Supplement table 3 was used for adult data only. Not all patients were ART naïve, but overall 94% were naïve to ART. Two small programs had low percentages of ART naïve patients, 69% among 29 patients and 48% among 23 patients; ^23^Only 1 out of the 12 programs was in urban setting, but it accounts for 42.0% of the total sample size; ^24^At 12 months; ^25^In non-adherent group and in adherent group;^26^No multivariate regression but univariate association was done;^27^Forty one deaths in passive follow–up group and 124 deaths in active follow–up group;^28^5.5/100PY in active follow–up group and 2.7/100PY in passive follow–up group.
