# Supplementary material for: Early Mortality in Adults Initiating Antiretroviral Therapy (ART) in Low- and Middle-Income Countries (LMIC): A Systematic Review and Meta-Analysis
Source: PLoS One. 2011 Dec 29;6(12):e28691. doi: 10.1371/journal.pone.0028691 (PMC3248405; doi:10.1371/journal.pone.0028691)
Supplement: Table S3 — Studies assessing independent risk factors for early mortality organized by measure of association and by region. Ωonly those measures of association reaching statistical significance in multivariate analysis are included; BMI = Body Mass Index. All BMI values are baseline and in kg/m2 unless mentioned otherwise; CD4 = CD4 cell count. All CD4 counts are baseline and in cells/mm3 unless mentioned otherwise; Clinical staging = World Health Organization (WHO) Clinical staging unless mentioned otherwise; Hb = Hemoglobin, Hemoglobin values are baseline and in gm/dL unless mentioned otherwise; Age is in years unless mentioned otherwise; Viral Load = HIV–1 RNA plasma concentration, Viral load is in log copies/ml unless mentioned otherwise; TLC = Total leukocyte count, TLC is cells/mL unless mentioned otherwise; CTX site = Cotrimoxazole treatment site; TB = Tuberculosis; KS = Kaposi's Sarcoma; ART = Anti-retroviral therapy; HAART = Highly active antiretroviral therapy; F = Female; M = Male. (DOCX) [file pone.0028691.s003.docx]

| **Study** | **Year** | **Variables Assessed** | **Strength of Association (Measure of Association^Ω^)** | | | | | | | | | |
| --- | --- | --- | --- | --- | --- | --- | --- | --- | --- | --- | --- | --- |
|  |  |  | **BMI** | **BaselineCD4 Count cells/mm^3^** | **Clinical Staging** | | **Hb** | | **Age** | **HIV–1RNA Plasma Concentration** | **Sex** | **Others** |
| **Hazard Ratio (HR)** | |  |  |  |  | |  | |  |  |  |  |
| **Sub-Saharan Africa** | |  |  |  |  | |  | |  |  |  |  |
| Alemu | 2010 | Age, Sex, CD4, Clinical staging, Hb, Weight, CTX prophylaxis, facility | - | - | ^1^Real-case model:  Stage 1/2 1.00(HR)  Stage 4 5.13(HR)  Worst-case model:  Stage 1/2 1.00(HR)  Stage 4 3.69(HR) | | ^1^Real-case model:  <=10 1.00(HR)  >10 0.39(HR)  Worst-case model:  <=10 1.00(HR)  >10 0.64(HR) | | - | - | - | CTX use  Real-case model:  Absent 1.00(HR)  Present 0.14(HR)  Worst-case model:  Absent 1.00(HR)  Present 0.38(HR)  Baseline weight (kg)  Worst-case model  <=40 1.00(HR)  >=60 0.26(HR) |
| Alibhai | 2010 | Age, Sex, CD4, Marital status, Education, Occupation, | - | 0.95 (HR)^2^ | - | | - | | - | - | - | Education: None 1.00 (HR)  Primary 0.42(HR) |
| Palombi | 2009 | BMI, CD4, Clinical staging, Hb, Age, Sex, Adherence, Calendar year | Model 1: 0.91(HR)^3^  Model 2: 0.92(HR) | Model 1: 0.80 (HR)^4^  Model 2: 0.84 (HR) | Model 2:  Stage 1/2 1.00(HR)  Stage 3/4 1.78(HR) | | Model 1: 0.79 (HR)^5^  Model 2: 0.80 (HR) | | -  - | -  - | ^6^Model 1:  M 1.00 (HR)  F 0.51(HR)  Model 2:  M 1.00 (HR)  F 0.45 (HR) | Model 1:  Attendance to visits > 95% 0.74 (HR)^7^  Model 2:  Calendar year, per 1yr increase 0.87 (HR) |
| Abaasa | 2008 | Age, Sex, Education, Adherence | - | - | - | | - | | - | - | - | Adherence > 95 % 0.46 (HR)  Any education 0.67 (HR) |
| De Beaudrap | 2008 | BMI, CD4, TLC | Model 1: 0.84(HR)^3^  Model 2: 0.82(HR) | Model 1: 0.87 (HR)^8^ | - | | - | | - | - | - | TLC per 200cells/μl increment 0.80 (HR) |
| Bisson | 2008 | CD4, Hb, Sex | - | >100 1.00 (HR)  50-99 2.51 (HR)  <50 3.86 (HR) | - | | - | | - | - | F 1.00 (HR)  M 1.74 (HR) | - |
| Johannessen | 2008 | BMI, Clinical staging, Hb, Sex, TLC , ART start year, Platelet count | >18.5 1.00 (HR)  <16 2.12 (HR) | - | - | | >12(F) 1.00(HR)  >13(M)1.00(HR)  8-9.9 7.50(HR)  <8 9.20(HR) | | - | - | - | Platelet count:>150x10^9^/L 1.00 (HR)  <150x10^9^/L 2.30 (HR)  ART start year : 2003-04 1.00 (HR)  2005-06 0.40 (HR) |
| Laurent | 2008 | CD4 | - | >50 1.00 (HR)  <50 5.69 (HR) | - | | - | | - | - | - | - |
| MacPherson | 2008 | Weight , CD4, Clinical Staging , Sex | > 45kg 1.00 (HR)  <45kg 2.48 (HR) | >50 1.00 (HR)  <50 2.90 (HR) | Stage 1/2 1.00(HR)  Stage 3/4 1.59(HR) | | - | | - | - | F 1.00 (HR)  M 1.62 (HR) | - |
| **Study** | **Year** | **Variables Assessed** | **Strength of Association (Measure of Association^Ω^)** | | | | | | | | | |
|  |  |  | **BMI** | **BaselineCD4 Count cells/mm^3^** | **Clinical Staging** | | | **Hb** | **Age** | **HIV–1RNA Plasma Concentration** | **Sex** | **Others** |
| Mulenga^9^ | 2008 | Creatinine clearance | - | - | - | | | - | - | - | - | Creatinine clearance:  Normal 1.00 (HR); Mildly reduced 1.70(HR); Moderately reduced 2.30 (HR); Severely reduced 4.30 (HR) |
| Toure | 2008 | BMI, CD4, Clinical staging,Hb, Sex | >25 1.00 (HR)  18.5-25 0.93 (HR) <18.5 193(HR) | >150 1.00 (HR)  101-150 1.32 (HR)  51-100 1.63 (HR)  <50 2.72(HR) | Stage1/2 1.00(HR)  Stage 3/4 1.61(HR) | | | >10 1.00(HR)  <10 1.22(HR) | - | - | F 1.00 (HR)  M 1.52 (HR) | - |
| Karcher | 2007 | Clinical staging, Age, Adherence | - | - | No AIDS 1.00(HR)  AIDS 5.83(HR) | | | - | 1.06 (HR)^10^ | - | - | Adherence 2months^11^ 1.05 (HR) |
| Lowrance | 2007 | CD4, Clinical staging, Age, Sex, CTX prophylaxis,  TB | - | - | Stage 3 1.00(HR)  Stage 4 1.99(HR) | | | - | - | - | F 1.00 (HR)  M 1.54 (HR) | Non-CTX site 1.00 (HR)  CTX site 0.62 (HR) |
| Etard | 2006 | BMI, CD4, Hb | < 19 1.00 (HR)  >19 0.54 (HR) | < 200 1.00 (HR)  > 200 0.43 (HR) | - | | | < 10 1.00 (HR)  > 10 0.56 (HR) | - | - | - | - |
| Ferradini | 2006 | BMI, CD4 , Clinical staging, Sex, | >18.5 1.00 (HR)  <18.5 2.92 (HR) | >50 1.00 (HR)  <50 1.64 (HR) | - | | | - | - | - | F 1.00 (HR)  M 1.63 (HR) | - |
| Jerene | 2006 | Clinical staging, TLC | - | - | Stage2/3 1.00(HR)  Stage 4 2.40(HR) | | | - | - | - | - | TLC : >750 1.00 (HR)  <750 3.60 (HR) |
| Lawn | 2006 | CD4,Clinical staging, Age, Viral load, Sex | - | 0.62 (HR)^12, 13^ | Stage1/2/3 1.00(HR)  Stage4 2.78(HR)^13^ | | | - | - | - | F 1.00 (HR)  M 2.00(HR)^13^ | CD4 count at 4 months: 0.42 (HR)^12, 14^ |
| Stringer | 2006 | BMI, CD4,Clinical staging,Hb,  Adherence | >16 1.00 (HR)  <16 2.40 (HR) | >200 1.00 (HR)  50-199 1.40 (HR)  <50 2.20 (HR) | Stage1/2 1.00(HR)  Stage 3 1.80(HR)  Stage 4 2.90(HR) | | | >8 1.00(HR)  <8 3.10(HR) | - | - | - | Non – adherence:  <90^th^ percentile 1.00 (HR)  >90^th^ percentile 2.90 (HR) |
| Coetzee | 2004 | CD4, KS | - | 2·41 (HR)^12^ | - | | | - | - | - | - | Kaposi sarcoma 4.82 (HR) |
| Djomand | 2003 | CD4, Clinical Staging | - | >50 1.00 (HR)  <50 5.90 (HR) | Stage 1/2 1.00(HR)  Stage 3/4 3.20(HR) | | | - | - | - | - | - |
| **Asia** |  |  |  |  |  | | |  |  |  |  |  |
| Chasombat | 2009 | Weight, CD4, Clinical staging, Age, Sex, Hospital type | >60kgs 1.00 (HR)  50-59 1.53 (HR)  40-49 2.75 (HR)  <40 6.29 (HR) | >200 1.00 (HR)  50-99 1.61 (HR)  <50 2.43 (HR) | Asymptomatic 1.00(HR)  Symptomatic 1.41(HR)  AIDS 1.75(HR) | | | - | 15-29 1.00 (HR)  >40 1.24 (HR) | - | F 1.00 (HR)  M 1.96 (HR) | Hospital Type:  District/Community 1.00 (HR)  private 0.63 (HR)  university 0.70 (HR)  regional 0.70 (HR)  province/general 0.86 (HR) |
|  |  |  |  |  |  | | |  |  |  |  |  |
| **Study** | **Year** | **Variables Assessed** | **Strength of Association (Measure of Association^Ω^)** | | | | | | | | | |
|  |  |  | **BMI** | **BaselineCD4 Count cells/mm^3^** | | **Clinical Staging** | | **Hb** | **Age** | **HIV–1RNA Plasma Concentration** | **Sex** | **Others** |
| Madec | 2007 | BMI, CD4, Clinical staging, Age, HAART discontinuation, CTX prophylaxis, Fluconazole prophylaxis | >18.5 1.00 (HR)  <17 2.47(HR) | >100 1.00 (HR)  20-50 2.78 (HR)  <20 8.93 (HR) | Stage3 1.00(HR)  Stage 4 1.86(HR) | | | - | 30-45 1.00 (HR)  >45 1.83 (HR) | - | - | HAART discontinuation >1month:  No 1.00 (HR)  Yes 3.23 (HR)  CTX prophylaxis: No 1.00 (HR)  Yes 0.15 (HR)  Fluconazole prophylaxis: No 1.00 (HR)  Yes 0.50 (HR |
| **Americas** |  |  |  |  | |  | |  |  |  |  |  |
| Severe | 2005 | Weight , CD4, Clinical staging, | 3.30 (HR)^15^ | >50 1.00 (HR)  <50 1.60 (HR) | | No AIDS 1.00 (HR)  AIDS 2.10 (HR) | | - | - | - | - | - |
| **Multi -Regional** |  |  |  |  | |  | |  |  |  |  |  |
| Tuboi | 2009 | CD4, Clinical staging, Age (per ten years) | - | ^16^100vs.50 0.79(HR)  200vs.50 0.58(HR)  350vs.50 0.43(HR) | | No AIDS 1.00(HR)  AIDS 3.10(HR) | | - | 1.12(HR)^17^ | - | - | - |
| Brinkhof | 2008 | CD4^18^, Clinical staging^19^, Treatment access, Follow-up | - | >50 1.00(HR)  <25 3.34(HR) | | Stage 1/2 1.00(HR)  Stage 3/4 5.35(HR) | | - | - | - | - | Treatment access: Free 1.00 (HR)  Fee 4.64 (HR)  Follow-up: passive follow-up 1.00 (HR)  active follow-up 5.15 (HR) |
| Braitstein | 2006 | CD4, Clinical staging, Age, Sex, ART regimen, Free access to treatment, Use of generic drugs, Routine monitoring of virological response, TB clinic on site | - | <25 1.00(HR)  100-199 0.37(HR)  200-349 0.14(HR)  >=350 0.34(HR) | | Stage 1/2 1.00 (HR)  Stage 3/4 2.02 (HR) | | - | - | - | - | Free access to treatment: No 1.00 (HR)  Yes 0.25 (HR)  TB clinic on site: No 1.00 (HR)  Yes 3.76 (HR) |
| Calmy | 2006 | BMI, CD4, Clinical Staging,Hb, Sex | >18 1.00(HR)  <18 2.38(HR) | >200 1.00(HR)  15-49 2.54(HR)  <15 3.63(HR) | | Stage 1/2 1.00(HR)  Stage 3 2.07(HR)  Stage 4 3.86(HR) | | >10 1.00(HR)  8-9 1.48(HR)  <8 2.62(HR) | - | - | F 1.00(HR)  M 1.75(HR) | - |
| **Odds Ratio(OR)** | | | | | | | | | | | | |
| **Africa** |  |  |  |  |  | | |  |  |  |  |  |
| Marazzi | 2008 | BMI, CD4, Clinical staging, Hb, Age, Sex, Viral load | >20 1.00 (OR)  18-20 1.59 (OR)  <18 4.87 (OR) | >200 1.00 (OR)  <200 1.70 (OR) | Stage 1/2 1.00 (OR)  Stage 3/4 1.73 (OR) | | | >10 1.00 (OR)  8-10 1.51 (OR)  <8 2.10 (OR) | 1.03 (OR)^10^ | <4 1.00 (OR)  4-5 2.15(OR)  >5 2.00 (OR) | - |  |
|  |  |  |  | | | | | | | | | |
| **Study** | **Year** | **Variables Assessed** | **Strength of Association (Measure of Association^Ω^)** | | | | | | | | | |
|  |  |  | **BMI** | **BaselineCD4 Count cells/mm^3^** | | **Clinical Staging** | | **Hb** | **Age** | **HIV–1RNA Plasma Concentration** | **Sex** | **Others** |
| Zachariah | 2006 | BMI,CD4, Clinical stage | >18.5 1.00 (OR)  17-18.4 2.10 (OR)  16-16.9 2.40 (OR) <15.9 6.00 (OR) | >200 1.00 (OR)  <50 2.20(OR) | Stage 3 1.00 (OR)  Stage 4 2.10 (OR) | | | - | - | - | - |  |
| Weidle | 2002 | CD4, Viral load | - | >50 1.00 (OR)  <50 2.93 (OR) | - | | | - | - | - | - |  |

**Legend & Footnotes: ^Ω^** only those measures of association reaching statistical significance in multivariate analysis are included; **BMI**= Body Mass Index. All BMI values are baseline and in kg/m^2^ unless mentioned otherwise; **CD4**= CD4 cell count. All CD4 counts are baseline and in cells/mm^3^ unless mentioned otherwise; **Clinical staging=** World Health Organization (WHO) Clinical staging unless mentioned otherwise; **Hb**= Hemoglobin, Hemoglobin values are baseline and in gm/dL unless mentioned otherwise; **Age** is in years unless mentioned otherwise; **Viral Load**= HIV–1RNA plasma concentration, Viral load is in log copies/ml unless mentioned otherwise; **TLC**= Total leukocyte count, TLC is cells/mL unless mentioned otherwise; **CTX site**= Cotrimoxazole treatment site; **TB**= Tuberculosis; **KS**= Kaposi’s Sarcoma; **ART**= Anti-retroviral therapy; **HAART**= Highly active antiretroviral therapy; **F**= Female; **M**= Male;

^1^Real-case model used mortality data observed and worst-case model assumed those who were lost to follow-up were dead;^2^Change in Hazard ratio/Odds ratio for every 10 cells/mm^3^ increase in CD4 cell count;^3^Change in Hazard ratio/Odds ratio for every unit increment in BMI;^4^ Change in Hazard ratio/Odds ratio for every100 cells/mm^3^ increase in CD4 cell count;^5^ Change in Hazard ratio/Odds ratio for every 1gm/dl increase in Hb concentration;^6^Hazard ratios can be converted to F 1.00 M 1.96 in model 1 and F 1.00 M 2.22 in model 2; ^7^Adherence described by participants who attended >95% of their drug pickup appointments;^8^Change in Hazard ratio/Odds ratio for every 25cells/mm^3^ increase in CD4 cell count; ^9^For mortality at or before 90 days of therapy; ^10^Change in Hazard ratio/Odds ratio for every unit increase in age; ^11^Mean Cumulative Adherence after 2 months of therapy; ^12^Analyzed in50 cells/mm^3^ increments;^13^For early deaths (<4 months post –HAART);^14^For late deaths(>4 months post–HAART); ^15^For men <48.9kg and for women <43kg; ^16^Hazard ratios can be converted to 50 vs 100: 1.27(HR); 50 vs 200: 1.72(HR); 50 vs 350: 2.33(HR)^17^Change in hazard ratio/odds ratio for every 10 years increment in age;^18^Not measured in1404 participants; ^19^Not performed in 1004 participant
