# Supplementary material for: Early Mortality in Adults Initiating Antiretroviral Therapy (ART) in Low- and Middle-Income Countries (LMIC): A Systematic Review and Meta-Analysis
Source: PLoS One. 2011 Dec 29;6(12):e28691. doi: 10.1371/journal.pone.0028691 (PMC3248405; doi:10.1371/journal.pone.0028691)
Supplement: Table S4 — Qualitative assessment of included studies. 1Ascertainment bias was calculated from two parameters, a) Loss to follow–up mentioned and b) Active method for ascertainment of mortality. If loss to follow–up was mentioned 1 point was assigned and if there was an active method for ascertainment of mortality then 1 point was assigned. The scoring system was thus: 2 points- Low chance of ascertainment bias; 1 point- Moderate chance of ascertainment bias; 0 points– High chance of ascertainment bias; 2Cotrimoxazole/Tuberculosis prophylaxis; 3Adherence was evaluated in various methods such as assigning a care taker for the patient, prescribing medication for a standard duration and asking the patients to come with the completed kit, counseling the patients and their care takers at the initiation of antiretroviral therapy about the importance of being adherent to medication;4Confounder control was said to be adequate if multivariable analysis was performed; 5Losses to follow–up was defined in various forms by each study and was accounted by different means such as making phone calls, home visits, attendance from patient registers etc; 6Adherence counseling was done to patients/patient guardian at the time of initiation of ART but adherence was never measured during the follow–up; 7211(3.8%) were lost to follow–up after 1stART visit while 880(16%) were lost to follow–up later on. (DOCX) [file pone.0028691.s004.docx]

| **Study** | **Year** | **Chance of Ascertainment Bias^1^** | **Disclosure of Prophylaxis^2^** | **Mention of Adherence^3^** | **Confounder Control^4^** | **Accounting for Loss to Follow–up^5^** |
| --- | --- | --- | --- | --- | --- | --- |
| **Sub-SaharanAfrica** | |  |  |  |  |  |
| Alemu | 2010 | Moderate | Yes | Yes | Yes | Yes |
| Alibhai | 2010 | Moderate | No | Yes | Yes | Yes |
| Palombi | 2009 | Low | No | Yes | Yes | Yes |
| Rougement | 2009 | Moderate | No | Yes | No | Yes |
| Sanne | 2009 | Moderate | No | Yes | No | Yes |
| Abaasa | 2008 | Moderate | No | Yes | Yes | Yes |
| Banda | 2008 | Low | No | No | No | Yes |
| De Beaudrap | 2008 | Low | No | No | Yes | Yes |
| Bisson | 2008 | Moderate | No | No | Yes | Yes |
| Boulle | 2008 | Moderate | No | No | No | Yes |
| Bussman | 2008 | Moderate | No | Adherence counseling done | No | Yes |
| Hoffmann | 2008 | Moderate | No | No | No | No |
| Johannessen | 2008 | Moderate | No | No | No | No |
| Laurent | 2008 | Moderate | Yes | No | Yes | No |
| MacPherson | 2008 | Low | No | No | Yes | Yes |
| Marazzi | 2008 | Low | Yes | No | Yes | Yes |
| Mulenga | 2008 | Moderate | No | No | Yes | Yes |
| Mzileni | 2008 | Low | No | No | No | Yes |
| Nakanjako | 2008 | Moderate | No | Adherence counseling done^6^ | No | Yes |
| Toure | 2008 | Moderate | Yes | Yes | Yes | Yes |
| Yu | 2008 | High | No | No | No | No |
| Karcher | 2007 | Low | No | Adherence counseling done | Yes | Yes |
| Lowrance | 2007 | Low | Yes | Yes | Yes | Yes |
| Makombe | 2007 | High | No | No | No | Yes |
| Makombe | 2007 | Moderate | No | No | No | Yes |
| Bekker | 2006 | Low | No | Adherence counseling done | No | Yes |
| Etard | 2006 | Low | No | No | Yes | Yes |
| Ferradini | 2006 | Moderate | No | No | Yes | Yes |
| Jerene | 2006 | High | No | No | Yes | No |
| Lawn | 2006 | Moderate | Yes | No | Yes | Yes |
| Libamba | 2006 | Moderate | No | No | No | Yes |
| Stringer | 2006 | Moderate | Yes | Adherence counseling done | Yes | No |
| Zachariah | 2006 | Low | Yes | No | Yes | Yes |
| Bourgeois | 2005 | Moderate | No | No | No | Yes |
| Wester | 2005 | Low | Yes | Yes | Unclear | Yes |
| Coetzee | 2004 | Moderate | No | Adherence counseling done | Yes | Yes |
| Djomand | 2003 | High | No | No | Yes | No |
| Weidle | 2002 | Low | No | No | Yes | Yes |
| **Study** | **Year** | **Chance of Ascertainment Bias^1^** | **Disclosure of Prophylaxis^2^** | **Mention of Adherence^3^** | **Confounder Control^4^** | **Accounting for Loss to Follow–up^5^** |
| **Asia** |  |  |  |  |  |  |
| Ruan | 2010 | Moderate | No | Yes | No | No |
| Chasombat | 2009 | Moderate | Yes | No | Yes | Yes |
| Morineau | 2009 | Low | No | No | No | No |
| Ferradini | 2007 | Moderate | No | Yes | No | Yes |
| Madec | 2007 | Moderate | Yes | No | Yes | Yes |
| **Americas** |  |  |  |  |  |  |
| Corey | 2007 | Low | Yes | No | No | No |
| Severe | 2005 | Moderate | No | No | Yes | Yes |
| **Multi-Regional** |  |  |  |  |  |  |
| O’Brien | 2010 | Moderate | No | Yes | No | No |
| Tuboi | 2009 | Moderate | No | No | Yes | Yes |
| Brinkhof | 2008 | Low | No | No | Yes | Yes^7^ |
| Braitstein | 2006 | Low | No | No | Yes- only those on active follow-up | Yes |
| Calmy | 2006 | Moderate | No | Yes | Yes | Yes |

**Legend: ^1^**Ascertainment bias was calculated from two parameters, a) Loss to follow–up mentioned and b) Active method for ascertainment of mortality. If loss to follow–up was mentioned 1 point was assigned and if there was an active method for ascertainment of mortality then 1 point was assigned. The scoring system was thus: **2 points**- Low chance of ascertainment bias; **1point-** Moderate chance of ascertainment bias; **0 points**– High chance of ascertainment bias; **^2^**Cotrimoxazole /Tuberculosis prophylaxis; **^3^**Adherence was evaluated in various methods such as assigning a care taker for the patient, prescribing medication for a standard duration and asking the patients to come with the completed kit, counseling the patients and their care takers at the initiation of antiretroviral therapy about the importance of being adherent to medication;^4^Confounder control was said to be adequate if multivariable analysis was performed; ^5^Losses to follow–up was defined in various forms by each study and was accounted by different means such as making phone calls, home visits, attendance from patient registers etc; ^6^Adherence counseling was done to patients/patient guardian at the time of initiation of ART but adherence was never measured during the follow–up; ^7^211(3.8%) were lost to follow–up after 1^st^ART visit while 880(16%) were lost to follow–up later on.
